# Transcriptome changes during osteogenesis of porcine mesenchymal stem cells derived from different types of synovial membranes and genetic background

**DOI:** 10.1038/s41598-023-37260-4

**Published:** 2023-06-21

**Authors:** Shuaichen Li, Puntita Siengdee, Michael Oster, Henry Reyer, Klaus Wimmers, Siriluck Ponsuksili

**Affiliations:** 1 Institute of Genome Biology, Research Institute for Farm Animal Biology (FBN), Wilhelm-Stahl-Allee 2, 18196 Dummerstorf, Germany; 2 Chulabhorn Graduate Institute, Program in Applied Biological Sciences, Chulabhorn Royal Academy, Kamphaeng Phet 6 Road, Laksi, Bangkok 10210 Thailand; 3 Faculty of Agricultural and Environmental Sciences, University of Rostock, Justus-von-Liebig-Weg 6b, 18059 Rostock, Germany

**Keywords:** Agricultural genetics, Stem-cell differentiation

## Abstract

Synovial membrane mesenchymal stem cells (SMSCs) often serve as in vitro model for bone disease, but the molecular mechanisms driving osteogenesis in SMSCs from different donor cells of various sources and breeds remain unclear. In this study, porcine SMSCs isolated from adipose synovium (FP) and fibrous synovium (FS) of Angeln Saddleback (AS) and German Landrace (DL) were used to discover the signaling network change after osteogenic induction. During osteogenic differentiation, mineral deposition was first observed at day 14 and further increased until day 21. Transcriptional changes between day 1 and day 21 were enriched in several signaling pathways, including Wnt, PI3K-Akt, and TGF-beta pathway. Certain pathways related to osteogenesis, including osteoblast differentiation, regulation of bone mineralization, and BMP signaling pathway, were enriched at late time points, as confirmed by the osteogenic markers *ALPL*, *COL1A1*, and *NANOG*. A fraction of differentially expressed genes (DEGs) were found between FP and FS, while DEGs between AS and DL increased during the differentiation phase until day 7 and then decreased from day 14 to day 21. These genes are involved in several important signaling pathways, including TGF-beta, Wnt, and lipid-related signaling pathways, suggesting that SMSCs from these two breeds have different osteogenic capabilities.

## Introduction

Mesenchymal stem cells (MSCs) have great potential to differentiate into a number of cell lineages when appropriately stimulated, which is why MSCs are currently considered an important constituent of regenerative medicine and cell-based application. Various adult tissues can be used to isolate MSCs, e.g. adipose tissue, dental pulp, bone marrow, etc., which have different abilities to differentiate into osteoblasts, chondrocytes, and adipocytes under appropriate conditions^1^. De Bari et al. isolated MSCs from the synovium for the first time^2^. Since then, synovial membrane-derived mesenchymal stem cells (SMSCs) have attracted attentions due to their advantages such as accessible sources, high proliferation rate, low immunogenicity, and greater chondrogenic differentiation potential compared to MSCs from other sources^3^. Therefore, SMSCs are frequently serving as a useful in vitro model of bone and joint diseases^4^.

There are two sources of SMSCs in the synovial membrane: fibrous synovium (FS)-SMSCs and adipose synovium (also known as the infrapatellar fat pad) FP-SMSCs. Most studies have focused on the process of chondrogenesis using SMSCs, which have higher chondrogenesis potential and are more accessible in vitro^5–8^. SMSCs also showed superiority not only in chondrogenesis but also in osteogenesis, myogenesis, and tenogenesis, which would make them a good candidate for musculoskeletal tissue regeneration^9,10^. MSCs play an important role in the pathogenesis of osteoarthritis, identified in normal structures and diseased tissue^11,12^, but there is still little research on the role of SMSCs in this disease progression. However, SMSCs, which are known to differentiate well in cartilage, may be beneficial for regeneration in bone disease as well.

More than 90% of bone cells are made up of osteocytes, which play vital roles in various physiological processes both within and beyond the bone microenvironment^13^. Bone is a highly dynamic organ whose steady state is regulated by osteoclasts and osteoblasts^14^. Using SMSCs derived from FS and FP to study osteogenesis is still limited. Many reports have been published on SMSCs, emphasizing their phenotype, proliferative capacity, and immunomodulatory features^5,6,15,16^. Little attention has been paid to the transcriptome as an important link to explain the phenotypic outcome and to explore how gene regulation controls the differentiation of SMSCs. Moreover, pigs are considered a suitable animal model to study the efficacy of SMSCs transplantation, due to the similarities in size and cartilage-specific properties of joints between pigs and humans^4^. Therefore, understanding the molecular processes in the differentiation of porcine SMSCs would be beneficial for the study and application of SMSCs in bone and joint diseases.

Breed differences are also likely to affect the differentiation capability of MSCs as known from a number of species. It was observed that there were significant differences in the expression of MSC markers between standardbred and thoroughbred horses^17^, while the bone marrow-derived mesenchymal stem cells (BMSCs) collected from different dog breeds also showed different efficiency during in vitro differentiation^18^. Furthermore, the discrepancies in MSCs differentiation potential also occur in different pig breeds^19^. The latest study reported that the osteogenic capability of dental pulp MSCs and periodontal ligament MSCs collected from miniature pig breeds was inferior to that from domestic farm pig breed^19^. Similarly, our previous study also showed that there were transcriptional differences between FS-SMSCs and FP-SMSCs from Angeln Saddleback (AS) and German Landrace (DL) breeds, though cell morphology and surface marker expression had considerable commonalities^20^. It is well known that traditional breeds, like AS, are characterized by lower protein synthesis ability but higher carcass fat yields^21^, while these features might reflect in the transcriptional regulations during SMSCs differentiation. Therefore, in this study, we focus on the molecular changes along the osteogenic differentiation process when comparing SMSCs derived FS and FP of two pig breeds differing in growth performance (DL), and fat deposition (AS). In addition, the molecular changes along several time points (1, 7, 14 and 21 days) were investigated by comparing differentiated SMSCs with controls, i.e., SMSCs grown in standard medium at the respective time points.

## Results

### Characterization of porcine SMSCs during osteogenic differentiation

In our previous study, we observed that the cell populations in the first and second passages exhibited non-uniform cell morphology^22^. Specifically, the primary nucleated cell numbers obtained from the FP tissue were approximately 0.5-fold lower than those from the FS tissue. Other morphological variations, such as the presence of fat-like cells or epithelial-like cells, were slightly visible in all primary cultures. However, the cells eventually displayed similar fibroblast-like morphologies and became relatively homogeneous and uniform after passage 3 which was used in this study. In addition, a macrograph of alizarin red staining, along with bright-field microscopy, clearly demonstrated the morphological differences between differentiated and non-differentiated (control) SMSCs for each cell type. However, no significant differences were observed between different breeds and tissue sources. For confirm the stemness of SMSCs, the positive conjugated antibodies against CD90, CD105, CD44, integrin beta 1 (CD29), and the FITC‐ or PE‐ coupled mouse IgG and IgG2a kappa isotype controls and the negative conjugated antibodies against CD45, CD34, and their mouse IgG1 kappa isotype controls were used, as reported in our previous studies^22^.

**Figure 1:**
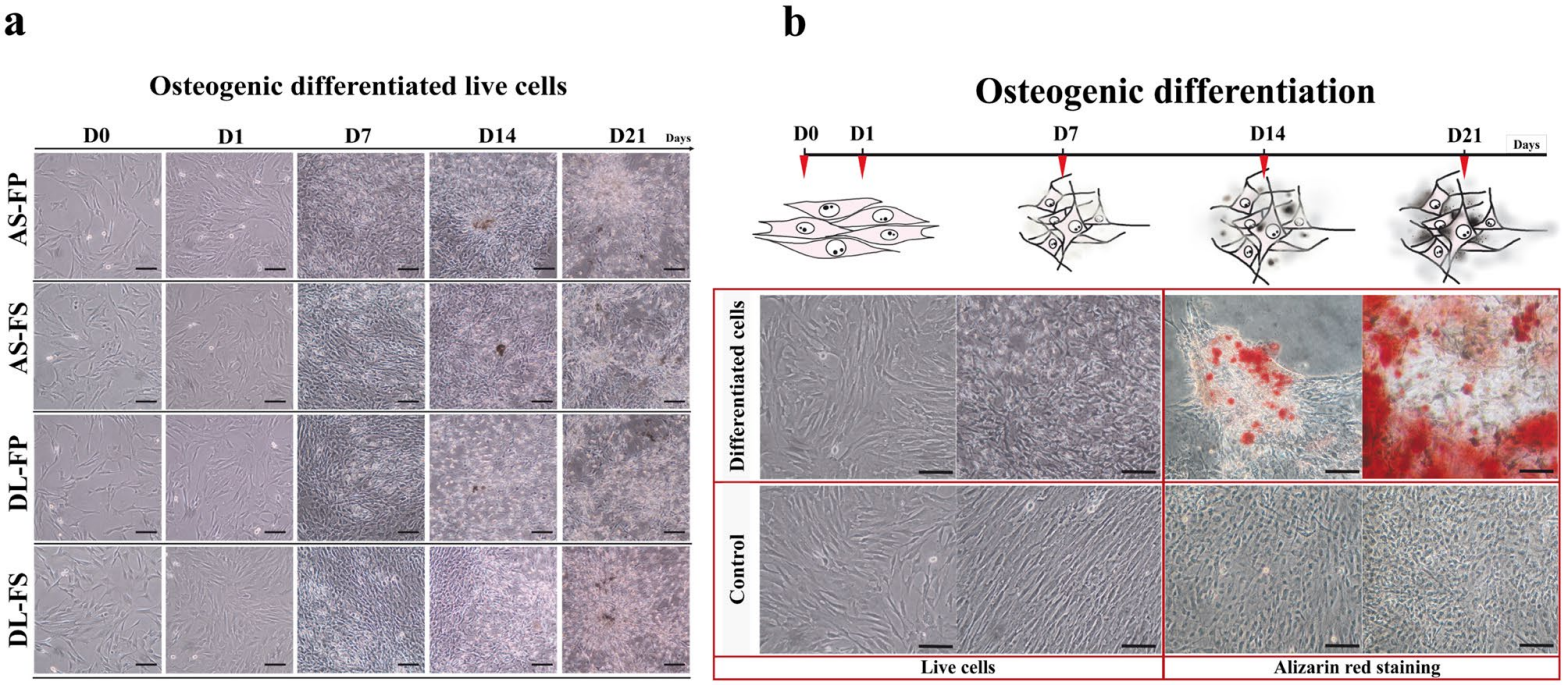
Osteogenic differentiation of SMSCs derived from different synovial tissue sites of both German Landrace (DL) and Angeln Saddleback (AS) pig breeds. (**a**) Live cell images of the undifferentiated and differentiated cells were taken at Day 0, Day 1, Day 7, Day 14 and Day 21. The late osteogenic stages starting at day 14 of differentiation after culturing in an osteogenic differentiation medium, cluster of osteocytes could form collagenous matrix and showed calcium mineralization deposits as brown–black lines or spots under a 20× objective phase-contrast microscope. Scale bars for all images: 100 μm. (**b**) Alizarin red staining analyses for the SMSCs during osteogenic differentiation. Cell morphology of the alizarin red stained cells after culturing in osteogenic differentiation medium for 14 and 21 days compared to the control group. Scale bars for all images100 μm.

All SMSCs obtained from two types of synovial locations and two different pig breeds presented spindle-shaped and plastic-adherent characteristics, while SMSCs cultured in ODM were successfully induced to undergo osteogenic lineage commitment. Brown-black lines or spots, indicating the existence of extracellular calcium accumulation, were visible in osteogenic-induced SMSCs between day 14 and day 21 (Fig. 1a). Alizarin red stained cells further demonstrated mineral deposits in these nodules, which were first observed at 14 days of differentiation and even more at day 21, whereas this was not the case for SMSCs cultured in CCM at the parallel time points (Fig. 1b).

### Comparison of the transcriptional profile of osteogenesis and control

**Figure 2:**
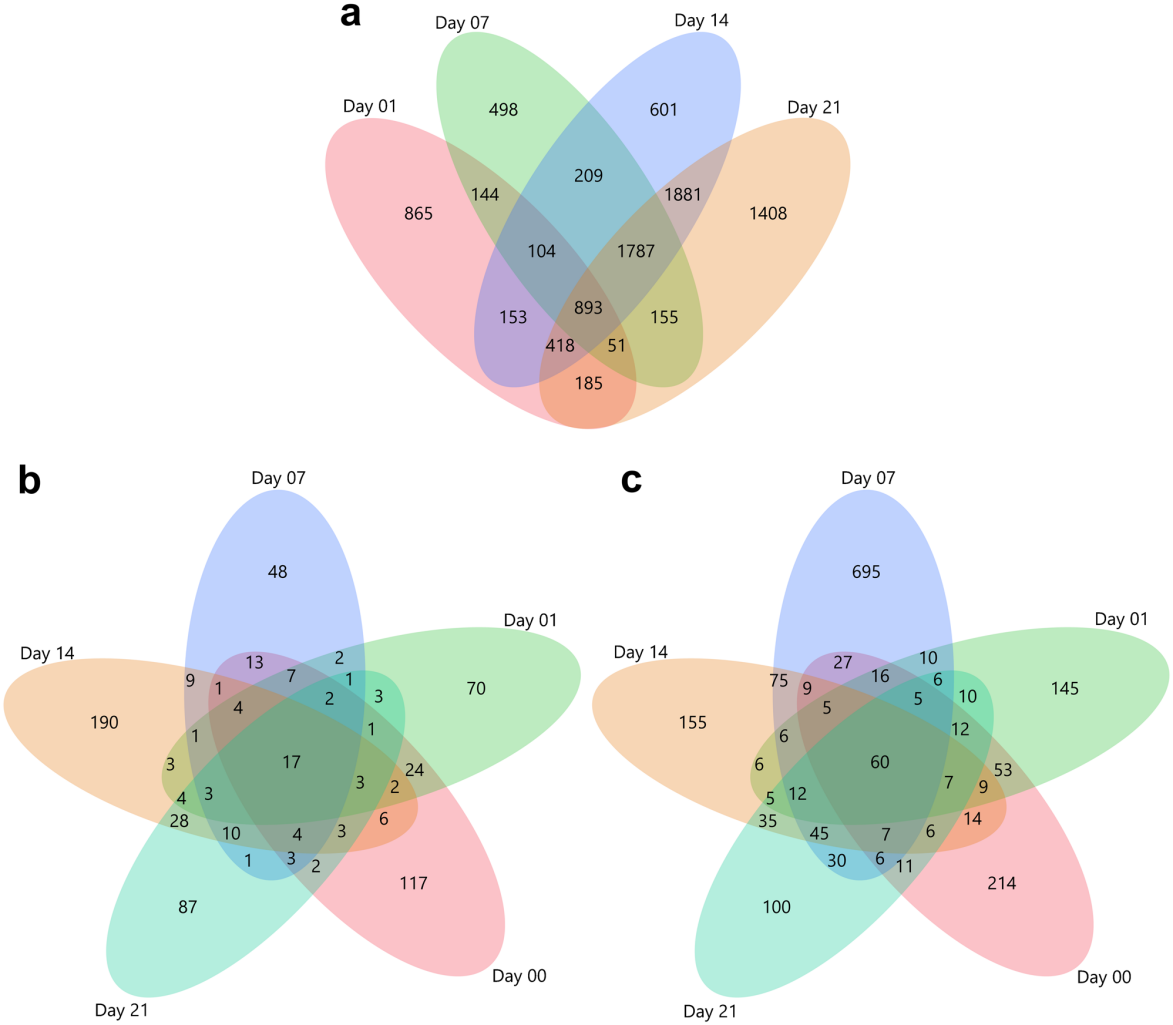
Venn diagrams of the number of specifically expressed transcripts at each time point when comparing (**a**) between control and osteogenic differentiation porcine SMSCs at day 1, 7, 14, and 21; (**b**) between porcine SMSCs harvested from adipose synovium (FP) and fibrous synovium (FS) prior to and after osteogenic induction at day 0, 1, 7, 14 and 21; (**c**) between porcine SMSCs derived from AS and DL prior to and after osteogenic induction at day 0, 1, 7, 14 and 21.

A total of 17,964 probe sets that passed quality control were used for further investigation. To investigate the regulation of osteogenic differentiation of SMSCs grown in ODM, we compared differentiated SMSCs with SMSCs grown in CCM at the same time point on day 1, day 7, day 14, and day 21 (Supplementary Table S1). The number of significant differentially expressed genes with FDR < 0.01 between osteogenic differentiation and SMSCs grown in CCM increased over time. During osteogenesis of porcine SMSCs, 2813 transcripts (1270 upregulated and 1543 downregulated) were significantly expressed at day 1, and this number further increased to 3841 transcripts (1698 upregulated and 2143 downregulated). This was followed by an even larger number of 6046 transcripts (2493 upregulated and 3553 downregulated) at day 14. At day 21, a total of 6778 differentially expressed transcripts were observed, including 2515 upregulated and 4263 downregulated. In addition, 865, 498, 601, and 1408 of these transcripts were exclusively expressed at day 1, day 7, day 14, and day 21, respectively, during the osteogenic differentiation of porcine SMSCs, as shown in Fig. 2a.

**Figure 3:**
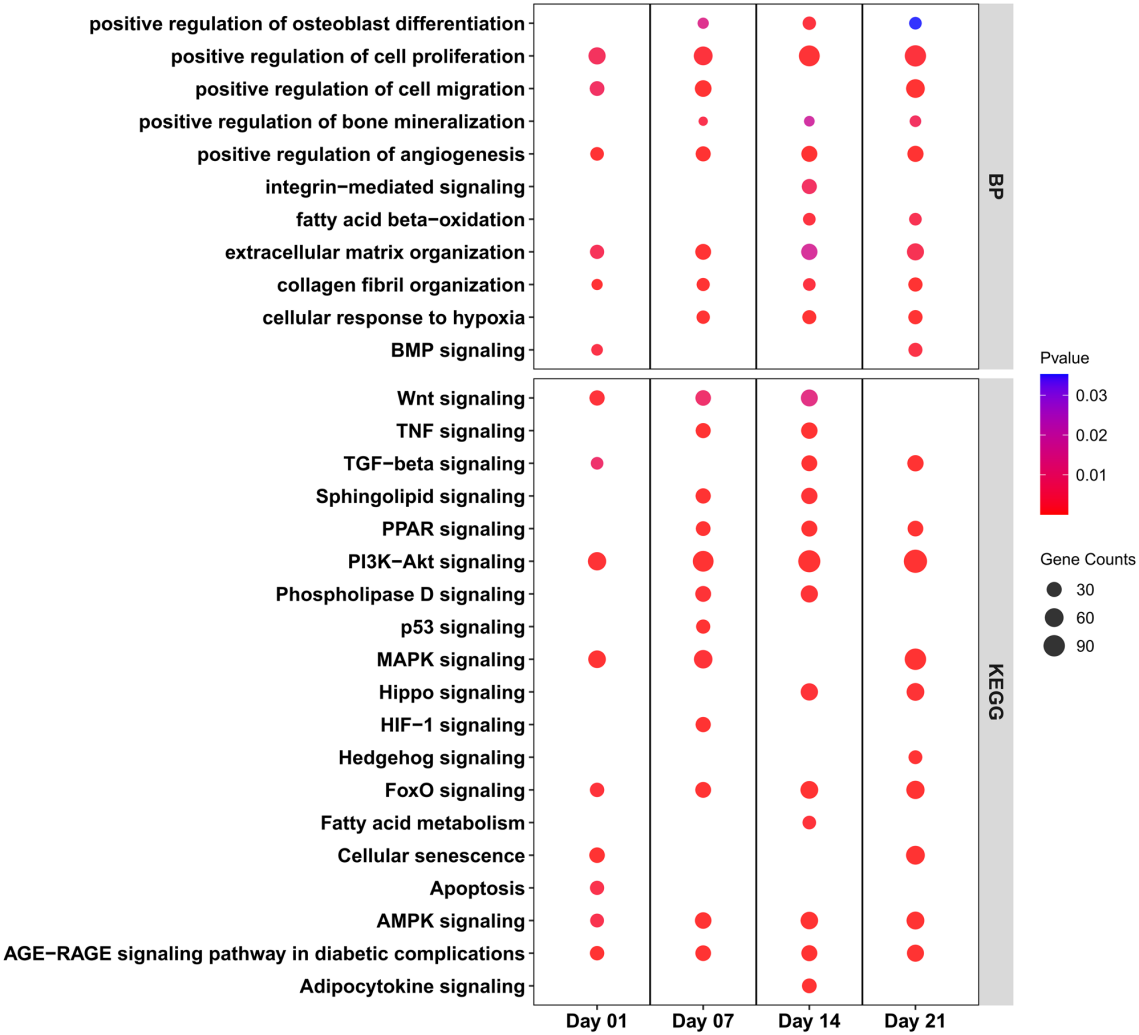
Biological process (**a**) and KEGG pathway (**b**) enrichment analysis of differentially expressed genes between control group and differentiation group. The dot size represents the number of genes involved in each biological process or KEGG pathways, while the dot's color indicates the *p*-value.

**Figure 4:**
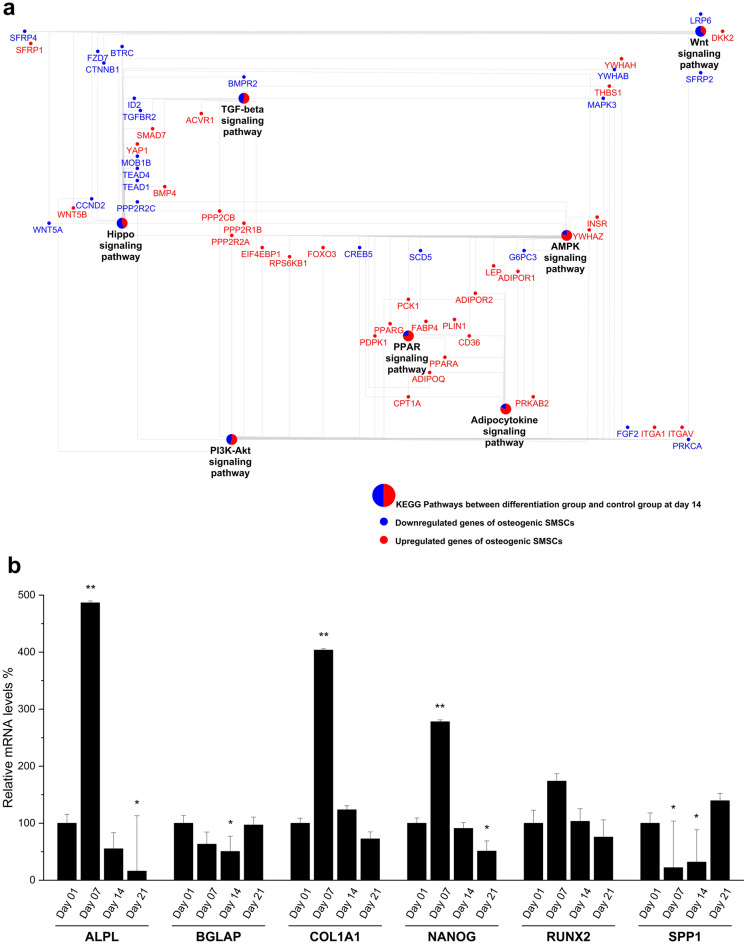
(**a**) Network of crosstalk pathways and their differentially expressed genes between control group and differentiation group at day 14. KEGG pathways are presented as pie charts in which the red and blue area represent the proportions of upregulated and downregulated genes of osteogenic SMSCs when comparing with that of control SMSCs. (**b**) Expression of osteogenic marker genes during osteogenesis at each time point compare to day 1 by qRT-PCR. Least squares means and standard errors were used and then transformed to relative expression level. *P < 0.05; **P < 0.01.

DEGs between differentiated SMSCs and SMSCs grown in CCM at the same time point on day 1, day 7, day 14, and day 21 were subjected to biological process (BP) and KEGG pathway analysis (Supplementary Table S2). Eight BP terms showed consistent enrichment across all time points. Most of these BPs belong to cell processes, such as cell proliferation, extracellular matrix organization, and angiogenesis process (Fig. 3). In addition, some signaling pathways, including TGF-beta receptor and canonical Wnt signaling pathway, were enriched at specific time points. Many osteogenesis-related terms in BP, including regulation of osteoblast differentiation, regulation of bone mineralization, and BMP signaling pathway, were enriched at late time points. In the analysis of KEGG pathways, we found eight pathways enriched across all time points, including PI3K-Akt signaling pathway, FoxO signaling pathway, AMPK signaling pathway, and AGE-RAGE signaling pathway (Fig. 3). DEGs enriched in PI3K-Akt signaling pathway, and stress pathways including cellular response to hypoxia or HIF-1 signaling pathways were also found at specific time points. The details of DEGs in some signaling pathways specific on day 14 were shown in Fig. 4a. During osteogenic differentiation, most upregulated transcripts clustered together in several metabolic processes, including TGF-beta signaling pathways, AMPK signaling pathways, PPAR signaling pathways, and Adipocytokine signaling pathway; on the other hand, most downregulated transcripts were found in signaling pathways such as Wnt signaling pathways and Hippo signaling pathways.

For the validation, 17 out of 19 (Supplementary Table S3) genes showed consistency between microarray and qRT-PCR data with Pearson’s coefficient of correlation (r) range from 0.6 to 0.9 with *p* value < 0.0001. Moreover, a dozen genes, linked with osteogenic differentiation, were not available in microarray data but observed in qRT-PCR data (Fig. 4b; Supplementary Table S3). During osteogenesis, the expression of *ALPL, COL1A1, RUNX2*, and *NANOG* was strongly increased at day 7, the early osteogenesis marker. In contrast, *BGLAP* and *SPP1* are more strongly expressed at the late time point of osteogenesis (day 21).

### Comparison between SMSCs derived from FP and FS

There is a small number of DEGs between SMSCs derived from FP and FS during osteogenesis (day 1, 7, 14, and 21) or prior to the osteogenic induction (day 0) (Supplementary Table S4). The number of significant differentially expressed transcripts was 209 at day 0, 147 at day 1, and 126 at day 7. The largest differences were noted on day 14 with 288 differentially abundant transcripts, followed by 172 transcripts on day 21. There were 117, 70, 48, 190, and 87 transcripts differentially expressed exclusively at day 0, day 1, day 7, day 14, and day 21, respectively (Fig. 2b). In view of a few DEGs, only a small fraction of pathways were observed when comparing different types of synovium. We noticed that many terms in BP and pathways in KEGG were enriched at day 0 and 14, such as cell migration, focal adhesion, MAPK signaling pathway, and PI3K-Akt signaling pathway (Supplementary Table S5).

Despite few pathways in comparison of SMSCs derived from different synovial sites, we further made a detailed comparison of some key genes associated with Wnt signaling pathway, osteogenic differentiation, and lipid metabolism by qRT-PCR (Supplementary Table S3). Among these genes, the expressions of many Wnt-related genes in SMSCs from FS were higher than that from FP, such as *AXIN2*, *WLS*, *WNT4*, *WNT10B*, *WNT11*, *FZD9*, and *LRP6*, except the Wnt receptor (*FZD7*) and two Wnt inhibitors (*FRZB* and *SFRP1*) with higher expression in FP. In addition, SMSCs derived from FP had greater expression in two osteogenic genes (*ALPL* and *SPP1*) and two lipid metabolism genes (*CEBPA* and *FABP4*), but lower expression in *NANOG*.

### Comparison between SMSCs derived from AS and DL

For the comparison of osteogenic differentiation between two breeds (Supplementary Table S6), the number of DEGs firstly slightly decreases at day 1, then peaks at day 7, followed by gradual decreases. Prior to differentiation (day 0), 461 transcripts were found, followed by 367 transcripts at day 1. Then, this number tripled at day 7 (1014 transcripts) mainly due to the higher abundance of many transcripts in AS. At day 14, the number of differentially expressed transcripts dropped to 456, with 309 upregulated and 147 downregulated transcripts. Subsequently, 357 transcripts were found at day 21 among which 149 were upregulated and 208 were downregulated. Besides, there were 214, 145, 695, 155, and 100 transcripts being identified to be specifically expressed at day 0, day 1, day 7, day 14, and day 21 (Fig. 2c).

**Figure 5:**
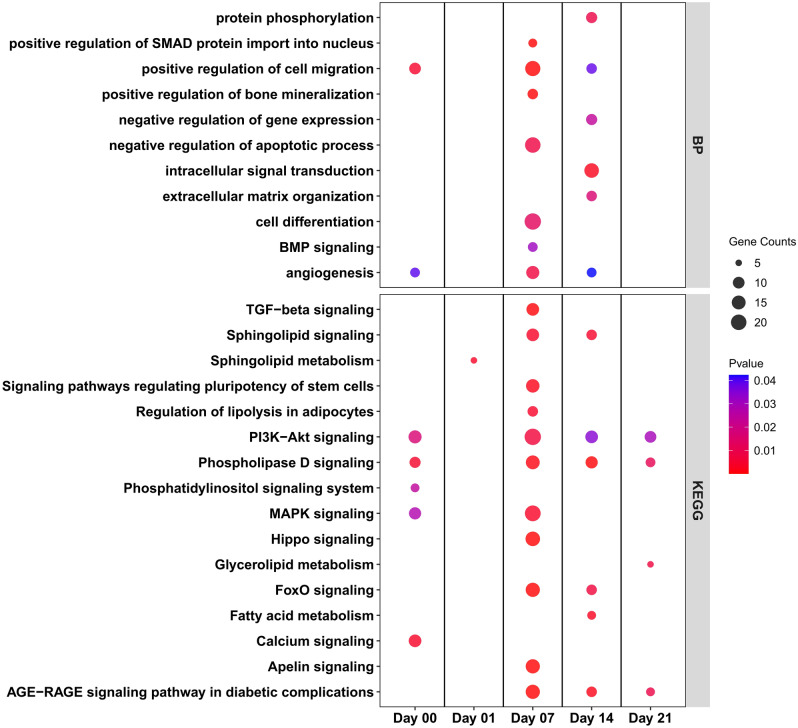
Biological process (**a**) and KEGG pathway (**b**) enrichment analysis of differentially expressed genes between porcine SMSCs derived from Angeln Saddleback (AS) and German Landrace (DL) after osteogenic induction at day 1, 7, 14 and 21 or undifferentiated state in SMSCs at day 0. The dot size represents the number of genes involved in each biological process or KEGG pathways, while the dot's color indicates the *p*-value.

**Figure 6:**
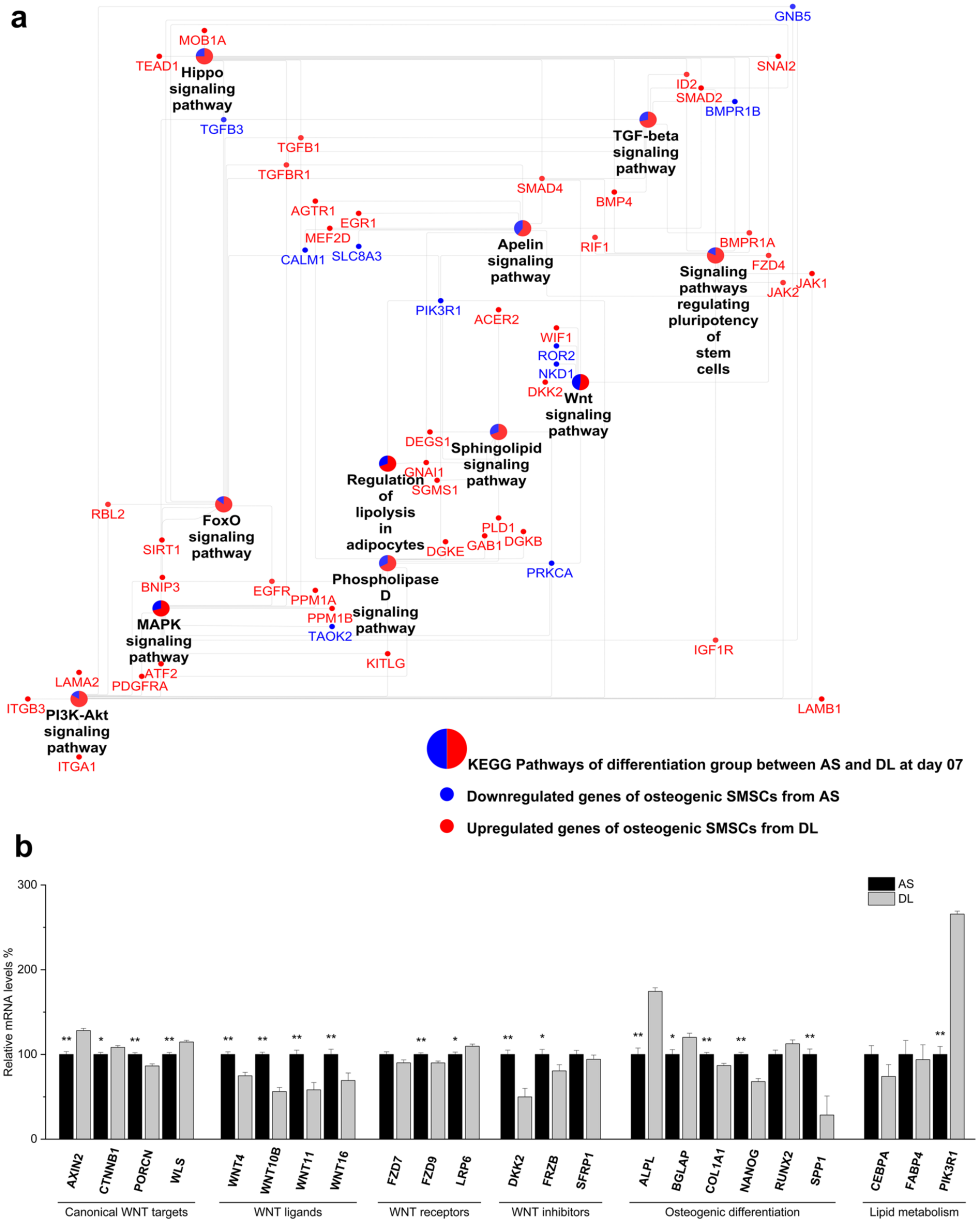
(**a**) Network of crosstalk pathways and their differentially expressed genes between Angeln Saddleback (AS) and German Landrace (DL) at day 7. KEGG pathways are presented as pie charts in which red area and blue area respectively represent the proportions of upregulated and downregulated genes of osteogenic SMSCs from AS when comparing with that from DL. (**b**) Expression of genes related to WNT signaling pathway, osteogenic differentiation, and lipid metabolism between AS and DL obtained by qPCR. Relative expression level were derived from least squares means and standard errors were used. *P < 0.05; **P < 0.01.

Both the analysis result of BP and KEGG showed that the differences related to osteogenic differentiation of porcine SMSCs derived from AS and DL mainly occur on day 7 and day 14 (Fig. 5; Supplementary Table S7). In the BP enriched terms, regulation of SMAD protein, regulation of bone mineralization, regulation of TGF beta receptor, BMP signaling pathway, and cell differentiation were found at day 7, while cell migration and angiogenesis were enriched both at day 7 and 14 (Fig. 5). In the KEGG enriched pathways, many osteogenesis-related pathways were observed at day 7, including TGF-beta signaling pathway, regulating of pluripotency of stem cells, and Hippo signaling pathway, while several lipid pathways were also found at day 7 and/or day 14, such as sphingolipid signaling pathway, regulation of lipolysis, phospholipase D signaling pathway, fatty acid metabolism, and apelin signaling pathway (Fig. 5). Part of crosstalk network at day 7 of KEGG pathways was shown in Fig. 6a. It can be seen that most genes in SMSCs from AS had higher expressions than that from DL during osteogenic differentiation. The results of qRT-PCR analysis also found higher gene expressions of Wnt ligands (*WNT4*, *WNT10B*, *WNT11*, and *WNT16*) and Wnt inhibitors (*DKK2* and *FRZB*) in AS (Fig. 6b; Supplementary Table S2). For osteogenesis-related genes, greater levels of *COL1A1*, *NANOG*, and *SPP1* were seen in AS, while the expressions of *ALPL* and *BGLAP* were lower when comparing SMSCs from DL.

### Longitudinal evaluation of SMSCs gene expression along the osteogenesis period

**Figure 7:**
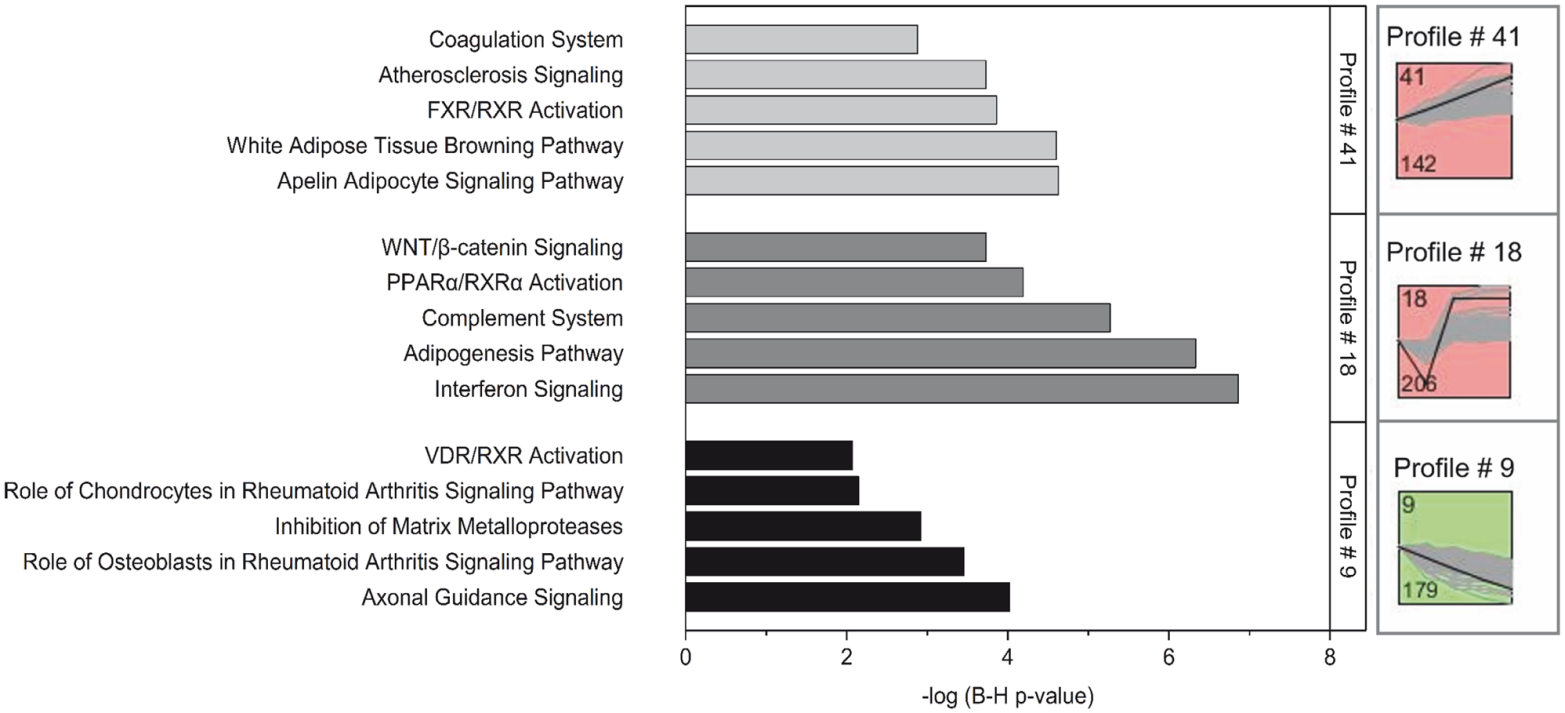
Top 5 canonical pathways enriched by IPA from selected STEM profiles. Profile # 9 display constantly increasing and profile # 41 display constantly decreasing mRNA abundances throughout osteogenic differentiation. Profile # 18 represents expressed genes specific to the late stage of osteogenic differentiation.

In the STEM analysis, ten profiles were considered significant. Among these profiles, 179 genes in profile # 9 and 142 genes in profile # 41 showed constant descending and ascending patterns over the whole differentiation period, respectively (Fig. 7). During the late stage of differentiation, 206 genes in profile # 18 remained high-level expression. These top three profile (9, 41 and 18) were selected for detailed analyses due to their clear differentiation-associated expression patterns over time relative to the overall experiment. The subsequent IPA analysis showed that inhibition of matrix metalloproteases (MMP) and rheumatoid arthritis-related pathway were observed in profile # 9, while adipogenesis-related pathways, like PPARα/RXRα activation, apelin adipocyte pathway, white adipose tissue browning pathways and FXR/RXR activation were found in profile # 41 and profile # 18. Also, Wnt/β-catenin signaling was enriched in profile # 18.

## Discussion

Various preclinical or animal studies have found that SMSCs harvested from humans, porcine and rodents have superior ability in cartilage repair or treating other bone and joint diseases^3,4^. However, there is still little knowledge about the biological role of SMSCs in these treatment processes. This is partly because the molecular mechanism of SMSC differentiation, in particular transcriptional regulation, is still unclear. Our previous study has characterized the morphological features and surface marker profiles of porcine SMSCs derived from two kinds of synovium (FS and FP) and two different breeds (AS and DL), which clearly showed that these SMSCs share similar cell morphologies and immune phenotypes but exhibit differences in molecular features^20^. Therefore, we further explored the transcriptional changes of porcine SMSCs during the osteogenic differentiation and the impact of different synovial sites and breeds on the porcine SMSCs differentiation.

In this study, we found that the number of DEGs between the osteogenic differentiation of SMSCs and the control group, where SMSCs were grown in CCM, increased continuously from day 1 to day 21, suggesting molecular and metabolic changes during the osteogenesis process. Many studies showed that most common GOs terms that were enriched in the early and late stages of MSC osteogenesis are associated with extracellular organization, cell communication, and adhesion^23^ as also found in our study including angiogenesis and extracellular matrix (ECM). Angiogenesis and ECM organization is seen as a key contributor to the osteogenesis process, as it plays crucial roles both in primary bone development and requires for mineralization^24,25^. The osteogenesis of bone marrow stromal cells (BMSCs) and adipose-derived mesenchymal stem cells (AMSCs) from human and porcine also showed an abundant expression of genes involved in vessel formation and ECM receptor interaction^26–28^.

Apart from the abovementioned pathways that have a general impact on the MSCs development, we also identified key genes and pathways that are widely considered as determinants of the fate of MSCs lineage commitment. The enrichment analysis revealed that GO terms related to ossification, osteoblast differentiation, and bone mineralization were particularly enriched at day 7 and/or day 14. For instance, *COL1A1* (collagen type I) is seen as an early marker of osteoblast^29^ and was significantly upregulated at day 7 in our study, in accordance with porcine and human BMSCs during osteogenic differentiation^26,30^. Osteoid (non-mineralized bone) consists of collagen type I (90%) and many non-collagenous proteins, while *SPP1* (osteopontin), *SPARC* (osteonectin) and *BGLAP* (osteocalcin) are the most abundant non-collagenous bone matrix proteins^31,32^. In our study, the expression of *SPARC* was increased during the osteogenic differentiation of porcine SMSCs from day 7 to day 14, which coincides with a previous study of porcine BMSCs^33^. In contrast to other studies using human and porcine MSCs^27,33^, we found by qRT-PCR that *BGLAP* was down-regulated at day 14 during osteogenic induction differentiation. It has been found that osteocalcin-deficient mice showed a higher capacity for bone formation^34^. A more recent study in Ocn^−/−^ mice also showed that Ocn is not involved in the regulation of bone formation but is important for bone strength^35^. Furthermore, one of the important osteoblastic phenotypes is the higher activity of ALPL (alkaline phosphatase). The expression of *ALPL* in differentiated SMSCs was significantly increased at day 7, indicating the process of differentiation of SMSCs into osteoblasts. The changes in these osteogenic genes indicate that early osteogenic lineage formation of porcine SMSCs occurs 7 days after in vitro induction.

The enrichment analysis results of KEGG pathways also showed that TGF-β and Wnt signaling pathways were mainly enriched in the middle and later periods of osteogenic differentiation, together with hippo signaling pathways. These pathways have also proven to be of importance for the osteogenic differentiation of MSCs^23,36,37^. Specifically, BMPs are part of TGF-β superfamily and involve in determining MSCs differentiation^37^. One of its members, BMP4, was highly expressed over the whole differentiation period in this study, while BMP4 is connected with high osteogenic activity and required for the formation and repair of endochondral bone^38,39^. Meanwhile, *ACVR1*, one of the BMPs type I receptors^40^, was also upregulated in the middle and late period of differentiation. TGF-β is deemed to promote early differentiation and matrix production of osteoblast progenitor cells, but also suppress later differentiation and mineralization^41^. In our study, the expression of *TGF-β2* in osteogenic SMSCs was upregulated at day 1 following a downregulation at day 21, while *TGF-β1* kept decreasing in the early and late periods of differentiation.

Wnt signaling was also recognized as one of the crucial pathway regulating osteogenesis^36,42^. In this study, six Wnt ligands were identified as differentially expressed in the results of microarray and qRT-PCR, while the changes of these ligands vary in time and directions. Similarly, different *FZD* subtypes showed different changes during the osteogenic differentiation, which may arise from different Wnts selectively binding to different FZD subtypes^43^. It is worth noting that *WNT5A* and *WNT5B* activity showed different regulations during the middle and late period of osteogenesis of porcine SMSCs, as also found in chondrocytes^44^. It has been reported that the roles of *WNT5A* and *WNT5B* are divergent when MSCs commit to being osteoblasts^45^. Surprisingly, the expression of canonical Wnt target genes, including *CTNNB* (β-catenin), *AXIN2*, and *LRP6*, continued to decrease from day 7 to day 21, as well as the expression of *PORCN* (porcupine) and *WLS* (Wnt Ligand Secretion Mediator) involved in the production and secretion of Wnt. In the same way, the downregulation of Wnt activators, such as *RSPO2*, and *KREMEN1*^46^, was observed in the late period of osteogenic differentiation. For the Wnt inhibitors, most of them, such as *DKK2*, *SFRP1*, and *APCDD1*^46^, were increased at day 14 and/or 21, except the expression of *FRZB*. These results showed the complex roles of Wnt signaling in the osteogenesis of porcine SMSCs, partly due to the presence of different subtypes of receptors and various agonists and antagonists that sometimes are inconsistent or have dual functions.

It is known that the master regulators that governed the direction of MSCs trilineage differentiation are *PPARG* in adipogenesis^47^, *RUNX2* in osteogenesis^48^ and *SOX9* in chondrogenesis. The osteogenic and adipogenic differentiation of MSCs is competing and reciprocal^47^. However, this might not be the case during the osteogenic differentiation of porcine SMSCs. A couple of lipid-related pathways, for instance, PPAR, fatty acid metabolism, and adipocytokine signaling pathways were enriched during the osteogenic differentiation of porcine SMSCs, but most of them were upregulated, especially the representative genes of *PPARG*. Although many studies considered it as an inhibitor of osteogenesis^49^, there are still studies of human BMSCs that showed the upregulated *PPARG* during osteogenic differentiation^50–52^. More surprisingly, no significant changes were found in the expression of *RUNX2*. This might be due to the fact that *RUNX2* can promote the differentiation of MSCs towards immature osteoblasts, but suppress osteoblast maturation^53^. Forcibly expressed *RUNX2* in osteoblasts led to failed osteoblast maturation and osteopenia^54^. Furthermore, it has also been reported that the ratios among *PPARG*, *RUNX2* and *SOX9*, instead of *RUNX2* alone, are key predictors of osteogenic potential of human BMSCs^50,52^. Taken together, these results demonstrated that the choice of the differentiation route of MSCs seems more complicated and requires for further investigation.

Anatomically, FP is sited between the joint capsule and FS, while FS is connected with joint internal structures and the musculoskeletal tissues^55^. Histologically, the intimal FS is formed with macrophage-like (type A) and fibroblast-like (type B) synoviocytes, while a large number of adipocytes constitutes FP together with a fraction of fibroblasts^56^. Even so, FP is seen as an adipose synovium instead of subcutaneous adipose tissue, since many studies confirmed that FP is an outgrowth of synovial tissue with regard to structure^56,57^. It is known that SMSCs can be isolated from both FS and FP, while the inherent properties of tissue-specific may affect the functions of MSCs^57,58^. In pig, FS-MSCs are able to generate the most mechanically functional tissue after 21 days of in vitro chondrogenic differentiation followed by 28 days of post in vivo implantation compared with FP-MSCs and BMSCs^8^. Hence, we also explored the transcriptomic differences of porcine SMSCs derived from FP and FS in this study.

Compared with the previous part of results, only a small fraction of DEGs and enriched pathways were identified, which may support the idea that FP and FS may be regarded as a morpho-functional unit^55^. Still, there were some interesting findings between FP and FS. During the process of osteogenic differentiation, the differences in several key pathways between two types of synovium were primarily found at day 14, including TNF-beta receptor, BMP, MAPK, and FoxO signaling pathway. The expressions of *COL1A1* and *NANOG*, upregulated in osteogenic SMSCs at day 7, were higher in FS-SMSCs at the same time point, which may indicate a slight difference between FS-SMSCs and FP-SMSCs during in vitro osteogenic differentiation.

To our knowledge, only a few studies explored the impact of animal breeds on the properties of MSCs, such as sheep, heifers, dogs, and horses^17,18,59,60^. However, these studies primarily focused on the differences in proliferation rate and cell surface markers of MSCs. It is still not clear to what extent the genetic characteristics of different animal breeds affect the underlying molecular mechanisms of MSCs during differentiation. It is well known that modern pig breeds such as DL have a high muscle content, while AS pigs, a traditional breed threatened with extinction, are characterised by a higher fat content, with the corresponding underlying metabolic differences between these breeds. These differences in fatty acids synthesis and lipid metabolism between two pig breeds not only reflect in slaughtered pigs^61^ but even display in newborn piglets^62^. Therefore, we present here a study of SMSCs from AS and DL pig breeds during osteogenic differentiation.

The results of the number of DEGs showed that the discrepancy of transcriptome profile between AS and DL increased during the differentiation period until day 7 and then decreased from day 14 to day 21. In particular, at day 7 and day 14, the time of active osteogenesis, most biological processes and signaling pathways were dominantly different between the breeds. The results of enrichment analysis confirmed that pathways related to bone mineralization and BMP signaling were enriched at day 7. More specifically, the expression of *BMP4* and *WNT11*, was higher in SMSCs derived from AS than from DL from day 7 to 14 after induction, while the upregulation of these two genes was also observed during the osteogenic differentiation of porcine SMSCs. This suggests that SMSCs derived from these two pig breeds have different osteogenic capabilities, as BMPs play important roles in regulating the proliferation and lineage-specific differentiation of mesenchymal stem cells^63^. The ossification-related genes, including *COL1A1, NANOG*, and *SPP1*, were more highly expressed in the SMSCs of AS, whereas the expression of *ALPL* and *BGLAP* was lower compared with the SMSCs of DL. Of interest is the *ALPL*, which encodes the enzyme alkaline phosphatase and is involved in bone mineralization, and *BGLAP*, also known as osteocalcin (OCN), which encodes the bone gamma carboxyglutamate protein responsible for binding to calcium and hydroxyapatite, the mineral component of bone. The new Ocn^−/−^ mice revealed that Ocn regulates bone quality not bone quantity or bone formation by aligning biological apatite (BAp) parallel to the collagen fibrils, which is important for bone strength^35^. Both genes were more highly expressed in SMSCs from DL, which may indicate greater mineralization and higher bone quality in this breed. A number of genes in AS were upregulated, involving MAPK signaling pathway, FoxO signaling pathway, TGF-beta signaling pathway, and Hippo signaling pathway, most of which were deemed to regulate the process of osteogenic differentiation^36^. We also observed that a set of upregulated genes involved in the regulation of SMAD protein were enriched at day 7, while SMAD proteins are bound up with TGF-beta and BMP signaling pathway in the regulation of bone homeostasis^64^. Therefore, these results may suggest that SMSCs derived from AS are more liable to differentiate into osteoblast than that from DL during in vitro osteogenic differentiation.

Previous studies found that there was a discrepancy with lipid-related genes or pathways in the muscle or adipose tissues of traditional and modern pig breeds^65^. Our earlier study also showed that the results of the original cell donor can be traced back to the specific characteristics of the breeds^20^. Although lipid-related pathways were not enriched prior to induction, the upregulation of several key genes at day 0 still disclosed genetic differences between AS and DL, such as *ADIPOQ* (adiponectin), *SMPDL3A* (sphingomyelin phosphodiesterase acid like 3A), and *PLIN2* (perilipin 2). *ADIPOQ* and *SMPDL3A* play a role in fat metabolism, while *PLIN2* is involved in the formation and maintenance of lipid droplets. The higher expressions of these obesity-associated genes were also observed in other fat-type pigs^66,67^. As osteogenic differentiation progressed, the most striking differences in lipid metabolism between SMSCs derived from AS and DL were noted at day 7 and 14 after induction, particularly enriched in sphingolipid signaling pathways, glycerolipid metabolism, apelin signaling pathway, and regulation of lipolysis in adipocytes. It is not surprising that most involved genes were higher in AS than in DL in the process. Growing studies showed that sphingolipid metabolism performs a role in the development of skeletogenesis^68^. It has been presumed that glycerolipid metabolism is a cause of differences in lipid deposition between different pig breeds^69^. For instance, umbilical cord blood MSCs also showed higher expression of glycerolipid during osteogenic differentiation^70^. Additionally, apelin is referred to as a member of adipokines that has been implicated in diabetes and obesity. However, it has a positive effect on the osteogenesis of human BMSCs^71^. In short, our study suggested that the differences of lipid metabolism during osteogenic differentiation can be traced back to the original breed of AS and DL and may influence the process of osteogenesis.

In summary, osteogenic differentiation induces significant changes in multiple molecular pathways before mineral deposition, as evidenced by alizarin red staining at day 14 and through day 21. These transcriptional changes primarily involve enriched signaling pathways, including Wnt, PI3K-Akt, and TGF-beta. Notably, late-stage time points exhibit enrichment of signaling pathways associated with osteogenesis, such as osteoblast differentiation, regulation of bone mineralization, and BMP signaling. Comparing different synovial types reveals a limited number of differentially expressed genes (DEGs), reflecting only a small portion of the signaling pathways. Importantly, the genetic background of donor MSCs plays a crucial role in osteogenesis, as evidenced by SMSCs derived from pig breeds with differ in growth performance (DL) and fat deposition (AS). Most of the molecular changes associated with the osteogenic differentiation of porcine SMSCs derived from AS and DL occur predominantly at day 7 and 14. These changes involve the regulation of bone mineralization, BMP signaling pathway, and Wnt signaling pathway. Notably, genes such as ALPL, involved in bone mineralization, and BGLAP, the mineral component of bone, exhibit higher expression in the DL groups, indicating differing osteogenic abilities between SMSCs derived from these two pig breeds. This evidence provides significant insights and serves as a crucial criterion for donor stem cell selection.

Overall, the potential of osteogenic differentiation of porcine SMSCs can serve the medical research on osteoarthritis, while functional analysis between different breeds might indicate differences in therapeutic application.

## Methods

### Culture and osteogenic differentiation of synovium-derived mesenchymal stem cell

Two types of synovial tissue were acquired from the stifle joint of piglets of two breeds. Male piglets of German Landrace (DL, n = 3) and Angeln Saddleback (AS, n = 3) breeds used in this study were 59 days of age. The SMSCs derived from adipose synovium (FP) and fibrous synovium (FS) were isolated, cultured, and induced to differentiate in osteogenic lineages as described previously^20^. The cells from passage 1 of previously frozen stocks were thawed and used in this study. In brief: FP- and FS-derived cells of three animals of the breeds DL and AS were grown from Passage 1 cryopreserved stocks by expanding the cultures in T75 cm2 culture flask at 37 °C with 5% CO2 in a humidified atmosphere. These SMSCs were already confirmed to express surface marker profiles indicative of stemness as reported in our previous studies^20^. We pooled the cells in passage 3 from three donor animals of each breed and each tissue and used these four pools in two replicates each at all time points. SMSCs at passage 3 were plated in a 24-well plate at 2 × 10^4^ cells/well in complete culture medium (CCM) (HG-DMEM, 4500 mg/L glucose Dulbecco’s modified Eagle’s medium, Gibco, New York, USA), supplemented with 10% FBS (Sigma-Aldrich, St Louis, USA) and 1% antibiotic/antimycotic solution. As SMSCs reached approximately 70% confluence, the medium of the osteogenic differentiation group was changed to the osteogenic differentiation medium (ODM), while the control group remained in the CCM. The ODM was comprised of HG-DMEM with 10% FBS, 5 µg/mL gentamycin, 100 nM dexamethasone (Sigma-Aldrich, St Louis, USA), 50 µM L-ascorbic acid (Sigma-Aldrich, St Louis, USA), and 10 mM β-glycerophosphate disodium salt hydrate (Sigma-Aldrich, St. Louis, USA). The mediums were replaced every 3–4 days.

Finally, osteogenic differentiation (ODM) was induced by culturing the cells for up to 21 days. The FP- and FS-derived cells from DL and AS at day 1, 7, 14 and 21 were collected used for further experiments. In addition, at each time point (0, 1, 7, 14 and 21 days) the non-differentiation cells (CCM) was done as control. In total 72 samples ([2 tissues × 2 breeds × 5 time points (0, 1, 7, 14, 21 days) for undifferentiated cells in CCM × 2 replicates)] + [2 tissues × 2 breeds × 4 time points (1, 7, 14, 21 days ) for differentiated cell in ODM × 2 replicates])were used.

### Histochemistry

In order to confirm osteogenic differentiation, 40 mM Alizarin-Red Staining Solution (Sigma-Aldrich, Taufkirchen, Germany) was used to detect a mineralized matrix as previously described^20^. In brief, adherent cell monolayers at each day both from control and osteogenic differentiation cells were washed with PBS and fixed with 4% formaldehyde in PBS for 30 min. After rinsing with distilled water, cells were covered with alizarin red for 30 min, washed until the supernatant was colorless, and then visualized by phase-contrast microscopy.

### RNA extraction, target preparation, and hybridization

Prior to osteogenic induction, SMSCs were collected and used as control group at day 0 for comparison between different breeds and tissues. Subsequently, at 1, 7, 14, and 21 days of cultivation, SMSCs in ODM and in parallel in CCM were collected. Total RNA of cells at each time point were isolated using TRI reagent (Sigma-Aldrich, Taufkirchen, Germany) and the RNeasy kit (Qiagen, Hilden, Germany) according to the manufacturer’s protocols. Both the quantity and purity of total RNA were measured using a NanoDrop ND-2000 spectrophotometer (Peqlab, Erlangen, Germany), while the integrity of RNA was determined via 1% agarose gel electrophoresis. All RNA samples were stored at − 80 ℃. Total RNA (500 ng) per sample was used for cDNA synthesis by the Affymetrix GeneChip WT PLUS Reagent Kit (Affymetrix, Santa Clara, CA, USA). The cDNA was fragmented and biotin-labeled via the Affymetrix GeneChip WT Terminal Labeling Kit (Affymetrix, Santa Clara, CA, USA), and then was hybridized on a genome-wide snowball array (Affymetrix, Santa Clara, CA, USA), containing 47,880 probe sets describing 17,964 annotated genes base on the Sscrofa11 genome [SNOWBALLs520824F]. After staining and washing, Affymetrix GCOS 1.1.1 software was used for scanning and processing the array data.

### Microarray data analysis

The pre-processed data analysis was processed using the Affymetrix Expression Console 1.4.1.46 software (Affymetrix). The robust multichip average (RMA) algorithm was used for normalization followed by the Log2-transformation of expression values. The detection above background (DABG) algorithm was carried out to filter present (expressed) genes. Probe sets present in less than 80% of the total samples were excluded from further analysis. Finally, a total of 20,036 probe sets passed the filter and were used for further analysis. The complete microarray datasets are deposited in the National Center for Biotechnology Information (NCBI) Gene Expression Omnibus (GEO) database (accession GSE219289: GSM6782094-GSM6782165).

The statistical analysis was performed using the MIXED procedure from the JMP genomics 10.1 software (SAS Institute, Cary, NC, USA). The model included the fixed effect of breeds, tissues, and state of SMSCs (osteogenic differentiation or control group at each collection day) and their interactions with breeds and tissues. The repeated statement of the Proc MIXED was used to consider and take advantage of the repeated measurements of each of the pools at all time points in CCM and ODM, respectively. The post hoc Tukey–Kramer test was used for multiple comparisons in all fixed effects. Then, probe sets with an adjusted *P*-value (FDR) less than 0.01 were regarded as differentially expressed genes (DEGs) according to the current annotation data^72^.

### Short time-series expression miner (STEM) for clustering expression pattern

In order to investigate the longitudinal gene expression data changes along osteogenic differentiation, the clustering algorithm implemented in the short time-series expression miner STEM (version 1.3.12)^73^ for co-expression profiling over each time point in SMSCs was chosen. The STEM clustering algorithm assigns each gene to the best-fitting profile (a specific co-expression profile) along the time points. A permutation test was applied to calculate the significance level of each profile based on the number of assigned genes versus the number of expected genes. In this study, the median of expression values from both breeds and tissues of two replicates per time point were submitted for the STEM analysis. The STEM clustering method was adopted with a filtering threshold at FDR < 0.05. Finally, gene symbols of top three significant profiles, consistent with the progress of the differentiation process, were submitted to IPA for the identification of canonical pathways based on the Ingenuity® Knowledge Base. Canonical pathway significance was tested at an adjusted *P-value* (Benjamini-Hochberg) < 0.05.

### Functional and pathway enrichment analysis

The results of DEGs in this study were divided into three parts for comparison: 1) DEGs between osteogenic differentiation group (ODM) and control group (CCM) from day 1 to day 21; 2) DEGs between FP and FS (SMSCs at day 0 and osteogenic-induced SMSCs from day 1 to day 21); 3) DEGs between AS and DL (SMSCs at day 0 and osteogenic-induced SMSCs from day 1 to day 21). All DEGs were uploaded to the Database for Annotation Visualization and Integrated Discovery (DAVID) and IPA to conduct functional and pathway enrichment analysis. For DAVID, gene ontology (GO) Biological Process (BP) enrichment, and Kyoto Encyclopedia of Genes and Genomes (KEGG) pathway enrichment were performed with default settings. Only terms or pathways with *P*-value < 0.05 were considered. For IPA, ingenuity canonical pathways were obtained by core analysis with default settings. Furthermore, pathways concerning cancer and infection will not be displayed and discussed in this study. The results of pathway enrichment were then visualized by ggplot2 package in R or ClueGO app (version 2.5.9) in Cytoscape (version 3.9.1).

### Quantitative real-time PCR (qRT-PCR) for validation and extension of microarray data

In order to validate the microarray expression data exemplarily and to supplement the set of genes represented on the microarray with those that are considered osteogeneic markers or belong to WNT signalling, qRT-PCRs were made of a total of 31 genes (Supplementary Table S8). Total RNA from the same samples was used. For cDNA synthesis, 200 ng RNA was mixed with 1 μL Reverse Transcription Master Mix (Fluidigm PN 100-6297) in 5 μL volume. The reaction was incubated at 25 °C for 5 min, 42 °C for 30 min followed by 85 °C for 5 min. The cDNA was used for qRT-PCR, which was conducted in the BioMark HD Real-time PCR System (Fluidigm, South San Francisco, CA) with a 48 × 48 dynamic array and an integrated fluidic circuit. Specific target amplification (STA) was done according to the manufacturer’s recommendations as described previously^20^. Pre-amplification was performed using PreAmp Master Mix (Fluidigm PN 1005581) at 95 °C for 2 min, followed by 10 cycles at 95 °C for 15 s and 60 °C for 4 min. The preamplification reaction was cleaned up using exonuclease I, followed by 10× dilutions of STA with DNA suspension buffer (TEKnova, PN T0221). The cycle threshold (Ct) was calculated by the software of the BioMark HD instrument. The Ct values were standardized using the geometric mean of two reference genes (*PPIA*, and *YWHAZ*) and quantifications were carried out using the 2^−ΔΔCt^ methods. Details of the primer sequences used in the study are shown in Supplemental Table S8. Correlation coefficient analysis between the microarray and qRT-PCR data was performed using JMP genomics 10.1 software.

### Ethics approval and consent to participate

This study was approved by the Animal Care Committee of the Research Institute for Farm Animal Biology and subject to the approved guidelines for safeguarding good scientific practice according to German Research Foundation (DFG). The measures were taken to minimize pain and discomfort and match with the guidelines laid down by the European Directive 2010/63/EU. The animals underwent no experimental treatment, diagnostic sampling, or any other intervention before slaughter. Animal handling was in line with applicable laws, relevant guidelines, and provisions for ethical regulations.

## Supplementary Information

Supplementary Tables.

## Data Availability

The expression datasets for this study can be found in the Gene Expression Omnibus public repository with the GEO accession number (GSE219289: GSM6782094–GSM6782165).
